# Assessment of Expert-Level Automated Detection of *Plasmodium falciparum* in Digitized Thin Blood Smear Images

**DOI:** 10.1001/jamanetworkopen.2020.0206

**Published:** 2020-02-28

**Authors:** Po-Chen Kuo, Hao-Yuan Cheng, Pi-Fang Chen, Yu-Lun Liu, Martin Kang, Min-Chu Kuo, Shih-Fen Hsu, Hsin-Jung Lu, Stefan Hong, Chan-Hung Su, Ding-Ping Liu, Yi-Chin Tu, Jen-Hsiang Chuang

**Affiliations:** 1 Taiwan AI Labs, Taipei City, Taiwan; 2 Taiwan Centers for Disease Control, Taipei City, Taiwan; 3 National Taipei University of Nursing and Health Sciences, Taipei City, Taiwan; 4 National Yang-Ming University, Taipei City, Taiwan

## Abstract

**Importance:**

Decades of effort have been devoted to establishing an automated microscopic diagnosis of malaria, but there are challenges in achieving expert-level performance in real-world clinical settings because publicly available annotated data for benchmark and validation are required.

**Objective:**

To assess an expert-level malaria detection algorithm using a publicly available benchmark image data set.

**Design, Setting, and Participants:**

In this diagnostic study, clinically validated malaria image data sets, the Taiwan Images for Malaria Eradication (TIME), were created by digitizing thin blood smears acquired from patients with malaria selected from the biobank of the Taiwan Centers for Disease Control from January 1, 2003, to December 31, 2018. These smear images were annotated by 4 clinical laboratory scientists who worked in medical centers in Taiwan and trained for malaria microscopic diagnosis at the national reference laboratory of the Taiwan Centers for Disease Control. With TIME, a convolutional neural network–based object detection algorithm was developed for identification of malaria-infected red blood cells. A diagnostic challenge using another independent data set within TIME was performed to compare the algorithm performance against that of human experts as clinical validation.

**Main Outcomes and Measures:**

Performance on detecting *Plasmodium falciparum*–infected blood cells was measured by average precision, and performance on detecting *P falciparum* infection at the image level was measured using sensitivity, specificity, and area under the receiver operating characteristic curve (AUC).

**Results:**

The TIME data sets contained 8145 images of 36 blood smears from patients with suspected malaria (30 *P falciparum*–positive and 6 *P falciparum*–negative smears) that had reliable annotations. For clinical validation, the average precision was 0.885 for detecting *P falciparum*–infected blood cells and 0.838 for ring form. For detecting *P falciparum* infection on blood smear images, the algorithm had expert-level performance (sensitivity, 0.995; specificity, 0.900; AUC, 0.997 [95% CI, 0.993-0.999]), especially in detecting ring form (sensitivity, 0.968; specificity, 0.960; AUC, 0.995 [95% CI, 0.990-0.998]) compared with experienced microscopists (mean sensitivity, 0.995 [95% CI, 0.993-0.998]; mean specificity, 0.955 [95% CI, 0.885-1.000]).

**Conclusions and Relevance:**

The findings suggest that a clinically validated expert-level malaria detection algorithm can be developed by using reliable data sets.

## Key Points

**Question:**

Can deep learning be used to develop an automated malaria detection algorithm?

**Findings:**

In this diagnostic study that used a 1-stage deep learning framework and benchmark data sets, the malaria detection algorithm achieved expert-level performance in detecting *Plasmodium falciparum* in thin blood smear images. The comparable performance between the algorithm and human experts was confirmed by a clinical validation study at the cell level and the image level.

**Meaning:**

The findings suggest that a clinically validated expert-level malaria detection algorithm could be used to accelerate the development of clinically applicable automated malaria diagnostics.

## Teaser

This diagnostic study assesses an expert-level detection algorithm for *Plasmodium falciparum*, a bacteria that causes malaria, using a publicly available benchmark image data set.

## Introduction

Malaria, a mosquito-borne disease caused by *Plasmodium* species, is a severe and reemerging global health issue despite years of effort in global malaria control. In 2017, an estimated 219 million cases of malaria and 435 000 malaria-related deaths occurred worldwide.^1^ Most patients were in the World Health Organization African region (92%) and South-East Asian region (5%). Although Taiwan has been certified malaria free for more than 5 decades, imported cases, mostly from Africa and Southeast Asia and caused by *Plasmodium falciparum*, still occur every year.^2^

The criterion standard for malaria diagnosis is microscopic examination.^3,4^ Thick blood smears are used for screening, whereas thin blood smears are used for confirming the species and measuring parasite density.^3^ However, conventional microscopic diagnosis is labor intensive and dependent on techniques and experience. This expertise is rare not only in resource-limited countries, where malaria poses a significant burden, but also in countries close to malaria elimination where the microscopists lack experience.^4^ Both situations prompted efforts to seek more efficient and accurate diagnostic tools. Economical and reliable rapid diagnostic tests (RDTs) can replace thick smears as a screening tool in resource-limited settings. However, RDTs provide insufficient information about species, life-cycle stages, and quantification of parasitemia, which are pivotal for clinical management.^3,4^ Therefore, researchers from engineering and computer science have performed extensive studies for an automated microscopic examination during the past decade.^5^ Nevertheless, progress toward a clinically applicable system was slow because of several challenges. First, the variety of staining methods and quality of smear preparation for microscopic blood smear images makes it difficult to devise universal features using traditional approaches. Second, it is hard to compare different algorithms because of different evaluation metrics and the absence of reference benchmark data. The difficulty of acquiring a large number of images with reliable annotations for public reference hinders the development of automated malaria diagnosis.

Traditional approaches to detecting malaria-causing organisms on thin smears involve a multistage approach of image preprocessing, red blood cell (RBC) segmentation, feature engineering, and classification of infected and noninfected RBCs.^5^ With the advent of deep learning, convolutional neural network (CNN)–based algorithms have achieved expert-level performance in the detection of pathologic characteristics in multiple medical image modalities,^6,7,8^ and the potential of applying deep learning on both thick and thin smears has been explored.^9,10,11^ Because thin smears can provide more clinical information, we aimed to assess high-quality image data sets of thin blood smears with expert annotations for public reference, develop a CNN-based algorithm to identify *P falciparum* infection automatically, and validate its performance in a clinical context.

## Methods

**Figure 1.**
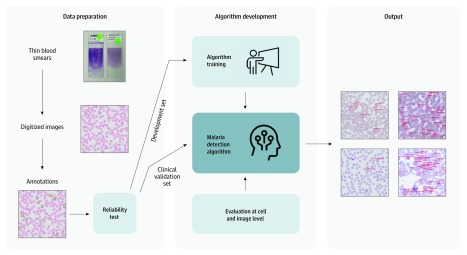
Overall Pipeline of the Malaria Detection Framework

For this diagnostic study, our framework began with the acquisition and digitization of thin blood smears, followed by algorithm training and validation at the cell and image levels (Figure 1). The study was approved by the institutional review board of the Taiwan Centers for Disease Control (CDC), which waived informed consent because the clinical samples were acquired from the deidentified biobank of the Taiwan CDC. The study followed the Standards for Reporting of Diagnostic Accuracy (STARD) reporting guideline.

### Data Preparation

Data were retrospectively acquired from ex vivo peripheral blood samples from patients suspected of having *P falciparum* infection by local physicians and public health workers who alerted the Taiwan CDC from January 1, 2003, to December 31, 2018. Laboratory diagnosis was made by microscopic examination and polymerase chain reaction.^12^ The details of enrollment of patients and sample collection are described in eMethods 1 in the Supplement.

We used virtual slide microscopes (VS120, Olympus Corp) to scan the blood smears and acquired pictures via digital cameras. The whole slide was sliced into grids of 2 × 2 mm. We then used the 100 × objective scope with oil immersion to scan slides into Olympus virtual slide images (.vsi). Olympus VS-ASW software was used to convert the .vsi files into jpeg files of 2048 × 2048 pixels. All digitized images were annotated by microscopists at the Taiwan CDC using an in-house annotation tool. For each image, 2 levels of annotation were made. At the cell level, the *P falciparum*–infected RBCs were annotated according to our annotation guideline, which defined the targets and guided the appropriate annotations (eMethods 2 in the Supplement). At the image level, infection status was inferred from cell-level annotations based on whether any bounding box of *P falciparum*–infected RBCs was identified.

All images in the development set were reviewed and annotated by an experienced microscopist (M.-C.K.) responsible for microscopic diagnosis of malaria at the Taiwan CDC for more than 45 years. Because of the variability among different microscopists,^13,14^ a reliability test subset was generated by randomly sampling 500 images from blood smears in the development set (19-20 images per smear) and was annotated by 2 other experienced microscopists (S.-F.H. and H.-J.L.), who worked in the parasitology laboratory at the Taiwan CDC for more than 5 years. Interrater reliability was assessed using mean F1 scores (the harmonic mean of precision and recall used as a measure of accuracy and reliability) among pairs of annotators at the cell level,^15^ and the percentage of agreement and the Fleiss κ were calculated for multiple raters at the image level. For the clinical validation set, all images were annotated by 3 expert microscopists (M.-C.K., S.-F.H., and H.-J.L.) at the Taiwan CDC. Interrater reliability was similarly measured as described above.

### Development of the Algorithm

The malaria detection algorithm was developed based on Retinanet, a 1-stage object detection neural network.^16^ The architecture consisted of a backbone network and 2 subnetworks, as in the original work,^14^ with a specially crafted loss function termed *focal loss*. The only modification in our architecture was that 1 fewer convolution layer was adopted for the backbone network. The backbone network was a feature pyramid network built on top of a CNN^17^ that was responsible for computing convolutional feature maps for input images. ResNet50 pretrained on the ImageNet data set was used for the backbone network.^18,19^ The first subnetwork is the object class subnet in the architecture of a CNN, which predicts labels given the output of the backbone network. The second subnetwork is the box subnet, which calculates regression on bounding box locations. The network was trained with the development set to identify the location of *P falciparum*–infected RBCs and to classify their stages.

An Adam optimizer with a learning rate of 1 × 10^−5^ and gradient clipping were used for training. The α was set to .25 and the γ to 2 for the focal loss. Our models were trained with a computer with an Intel Xeon E5-2630 v4 2.20 GHz central processing unit with a NVIDIA GeForce GTX 1080 Ti GPU 11-GB graphic card and 128 GB of RAM. We referenced our algorithm to the Keras implementation of Retinanet.^35^

### Clinical Validation

A diagnostic challenge was conducted to compare the performance of the algorithm against clinical laboratory scientists. Four practicing clinical laboratory scientists were recruited from 2 medical centers in Taiwan (Chang-Gung Memorial Hospital and Taipei City Hospital Zhongxing Branch and Zhongxiao Branch) to review the clinical validation set and to annotate *P falciparum*–infected cells. The scientists were trained for malaria microscopic diagnosis in a national reference laboratory at the Taiwan CDC and had 5 to 10 years of working experience.

### Statistical Analysis

#### Algorithm Evaluation

Algorithm performance was evaluated at the cell and image levels. At the cell level, whether bounding boxes identified by the algorithm matched ground truth bounding boxes was determined by a matching process (eMethods 3 and eFigure 1 in the Supplement). Our evaluation focused on 2 primary end points: malaria detection, defined as the detections of any malaria parasites regardless of life cycle stages, and ring form detection, the most common and characteristic stage in peripheral blood smears of *P falciparum*.^20^

Metrics conventionally used for object detection in computer vision were applied at the cell level, including precision-recall curves with average precision and free-response receiver operating characteristic (ROC) curves. At the image level, ROC curves were plotted and areas under the ROC curve (AUCs) were computed using the Python package Scikit-learn, version 0.18.1 (Python Software Foundation).^21^ The 95% CIs for ROC curves were estimated through 1000 iterations of bootstrap analysis on sensitivity and specificity with α = .05. Error rates of microscopic diagnosis were calculated by counting every mistake equally.

Two operating points were selected at the image level to further characterize the algorithm performance. The first operating point, corresponding to the point with the highest Youden index, reflects the optimal point of algorithm performance with high sensitivity. It was chosen because high sensitivity is a prerequisite for a potential screening tool.^22,23^ The second operating point, the high specificity operating point, which approximates the mean specificity of practicing microscopists in the clinical validation set, could further characterize our algorithm performance against the 4 practicing microscopists. The 95% CIs for the sensitivity and specificity at the 2 operating points were calculated to be exact Clopper-Pearson intervals.^24^ Two-sided 95% CIs were computed using the Python package StatsModels, version 0.8.0.

#### Sensitivity Analysis

Experiments were designed to evaluate the association of different labeling strategies with malaria detection. For the development set, 2 sets of meta-labels were processed and inferred from the raw annotations. For the first set, we pooled the labels of young trophozoite (ring form) and trophozoite into the category trophozoite, together with the remaining labels of schizont and gametocyte; for the second set, all labels regardless of their life-cycle stages were merged into the category malaria infection. These 2 sets of meta-labels were then used to train another 2 malaria detection models with similar configurations delineated above. Their performances were measured with the same metrics.

## Results

### Development and Characteristics of Data Sets

**Figure 2.**
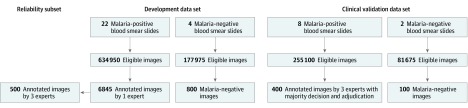
Workflow of Data Set Establishment

We established Taiwan Images for Malaria Eradication (TIME) data sets, which included 2 data sets: the development set for training and the clinical validation set for evaluating performance. Images in the development set were scanned from 26 blood smear slides (22 *P falciparum*–positive and 4 *P falciparum*–negative smears). Images in the clinical validation set were scanned from 10 blood smears (8 *P falciparum*–positive and 2 *P falciparum*–negative smears) (Figure 2). The development set included 6845 images from positive slides and 800 from negative slides that were randomly sampled from more than 812 000 raw scanned images. The clinical validation set included 400 images from positive slides and 100 from negative slides.

Of the 7645 images in the development set, 4402 images (57.6%) were identified with 21 220 *P falciparum*–infected cells annotated, among which the 2 most common life-cycle stages were young trophozoite (ring form; 80.1%) and trophozoite (10.9%), followed by gametocyte (1.3%) and schizont (0.2%). Of the 500 images in the clinical validation set, 400 (80.0%) were identified with 3061 *P falciparum*–infected cells. The most common life-cycle stages were ring form (2909 [95.0%]) and trophozoite (117 [3.8%]) (Table). Both data sets had considerable variation in parasite density and the level of touching cells (eFigure 2 in the Supplement). For *P falciparum*–positive smears, the median number of parasite-infected cells per image was 2 (range, 1-68) in the development set and 4 (range, 1-60) in the clinical validation set.

**Table.** Characteristics of the *Plasmodium falciparum–*Infected Blood Smear Image Data Sets^a^

Characteristic	Development Set	Clinical Validation Set
Blood smear slides	26	10
Annotators	1-3^b^	3
Images	7645 (100)	500 (100)
With *P falciparum*–infected cells	4402 (57.6)	400 (80.0)
Without *P falciparum*–infected cells	3243 (42.4)	100 (20.0)
Bounding boxes	21 220 (100)	3061 (100)
Ring form	16 992 (80.1)	2909 (95.0)
Trophozoite	2313 (10.9)	117 (3.8)
Schizont	35 (0.2)	1 (0)
Gametocyte	267 (1.3)	0
Indeterminate	1613 (7.6)	34 (1.1)
No. of bounding boxes per image, median (range)	2 (1-68)	4 (1-60)

^a^ Data are presented as number (percentage) of slides unless otherwise indicated.

^b^ One expert annotated all images, and 500 images selected from the whole set were also annotated by 2 additional experts.

### Interrater Reliability Test

At the cell level, the mean F1 score of the reliability test subset among pairs of experts was 0.924 (95% CI, 0.901-0.947), and the clinical validation set had an F1 score of 0.954 (95% CI, 0.938–0.970). At the image level, the percentage of agreement was 97.5% for the reliability test subset and 100% for the clinical validation set. The Fleiss κ among the 3 experts was 0.459 in the reliability test subset and 1.000 in the clinical validation set (eFigure 3 and eFigure 4 in the Supplement).

### Clinical Validation

**Figure 3.**
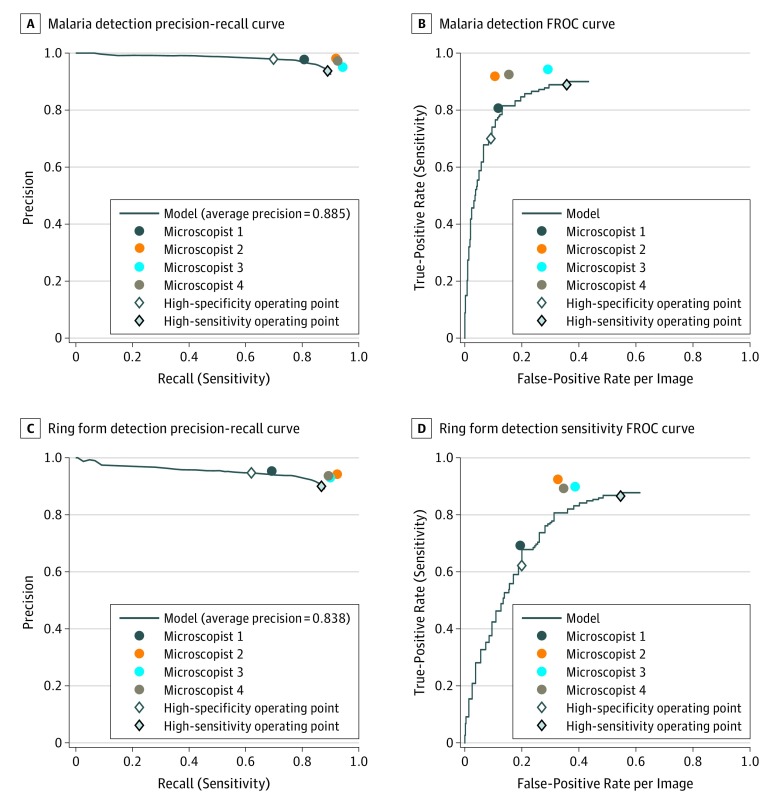
Performance of the Malaria Detection Algorithm at the Cell Level With Precision-Recall Curve and Free-Response Receiver Operating Characteristic (FROC) Curve

**Figure 4.**
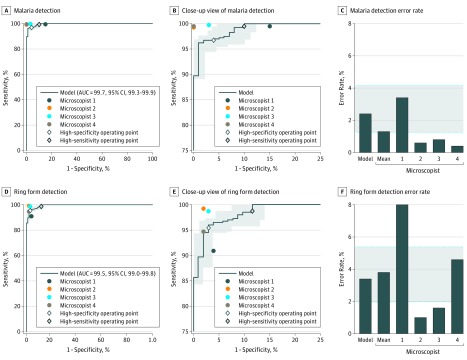
Performance of the Malaria Detection Algorithm at the Image Level With Receiver Operating Characteristic Curve and Error Rate The shaded area indicates the 95% CIs. AUC indicates area under the curve.

At the cell level, our algorithm achieved an average precision of 0.885 in detecting *P falciparum*–infected RBCs and 0.838 in detecting ring form *P falciparum*–infected RBCs (Figure 3). At the image level, our algorithm achieved an AUC of 0.997 (95% CI, 0.993-0.999) for malaria detection, which was comparable to experts’ performance (Figure 4A). At the high-sensitivity operating point, the sensitivity of our algorithm was 0.995 (95% CI, 0.982-0.999) and the specificity was 0.900 (95% CI, 0.824-0.951). At the high-specificity operating point, the sensitivity was 0.968 (95% CI, 0.945-0.983) and the specificity was 0.960 (95% CI, 0.901-0.989). For the error rate in identifying *P falciparum* infection, our algorithm achieved an error rate of 2.4% (95% CI, 1.3%-4.2%), showing no statistically significant difference compared with the mean error rate of microscopists (1.3%, 95% CI, 0.0%-3.5%).

For ring form detection at the image level, our algorithm had an AUC of 0.995 (95% CI, 0.990-0.998) (Figure 4B). At the high-sensitivity operating point, the sensitivity of our algorithm was 0.987 (95% CI, 0.971-0.996) and the specificity was 0.883 (95% CI, 0.805-0.938). At the high-specificity operating point, the sensitivity was 0.955 (95% CI, 0.929-0.973) and the specificity was 0.971 (95% CI, 0.917-0.994). This performance was comparable to experts' performance (mean sensitivity, 0.995; 95% CI, 0.993-0.998; mean specificity, 0.955; 95% CI, 0.885-1.025). The error rate was 3.4% (95% CI, 1.6%-5.4%), which was comparable to that of microscopists (3.8%; 95% CI, 0.0%-8.9%) (eFigure 5 and eFigure 6 in the Supplement).

### Sensitivity Analysis

On the clinical validation set, the model trained with meta-labels that pooled ring form and trophozoites as 1 category had an AUC of 0.987 (95% CI, 0.977-0.994) for malaria detection, whereas the model trained with meta-labels that pooled all parasites together as 1 category had an AUC of 0.994 (95% CI, 0.989-0.998) for malaria detection. The results indicated that the performance of these models trained with different labeling strategies were comparable to that of the original version.

## Discussion

In this study, we established benchmark image data sets of thin blood smears for malaria microscopic diagnosis. To our knowledge, this is the first intent to provide publicly available image data sets of microscopic blood smears with reliable annotation. Our data sets provide more than 8000 images with more than 24 000 annotated *P falciparum*–infected RBCs, encompassing a wide range of variations encountered in clinical settings. A CNN-based object-detection algorithm was developed with promising sensitivity and specificity to identify malaria detection and demonstrated performance similar to that of human microscopic experts (AUC of 0.997 and error rate of 3.4% for malaria detection and AUC of 0.995 and error rate of 2.4% for ring form detection).

From data set generation to algorithm development, our framework was designed with clinical applicability in mind. Previously released data sets typically consisted of images of individually segmented RBCs, designed for the 2-stage approach in which researchers segmented individual RBCs from microscopic images and then developed algorithms to classify them.^11,25,26,27^ However, touching cells on thin smears makes accurate RBC segmentation challenging. We designed our data sets for the development of 1-stage detection algorithms by providing images directly acquired from slides without additional preprocessing or segmentation. In this manner, we bypassed the segmentation problem and minimized artifacts resulting from human-designed preprocessing, ensuring the highest fidelity of the images with respect to the clinical reality. Retinanet was adopted as our 1-stage detector for its better performance, simpler structure, and previous application to other clinical contexts.^28,29^ Compared with the 2-stage approach, our algorithm demonstrated encouraging performance in dealing with touching blood cells. Furthermore, because the input images required no preprocessing, we minimized the computation cost both in hardware and processing time, ensuring rapid smear-to-diagnosis turnaround time.

For image annotation, previous data sets were usually annotated by only 1 expert,^27,30,31^ and the annotations might be susceptible to errors and biases because of the varied training protocol and experience of the individual annotator.^13,14^ To overcome this challenge, we recruited multiple microscopists and standardized the annotation process. In addition, because the reliability test showed a moderate to high level of agreement among our experts’ annotations at the cell and image levels, we are confident of the reliability of the annotations in our data sets. Furthermore, to our knowledge, this study was the first attempt to validate the algorithm performance against practicing microscopists in a clinical context. The expert-level detection algorithm demonstrated the potential to automate the microscopic examination, providing malaria diagnosis once a blood smear was made and photographed. Beyond streamlining the diagnosis workflow, our algorithm presents an opportunity to preserve and commoditize the increasingly scarce expertise in microscopic diagnosis, especially for countries close to malaria elimination but still dealing with imported cases related to international travel.

Given that malaria burden is greatest in countries with limited resources, we envision deploying a mobile application of our algorithm for unskilled workers with minimum equipment requirements.^25,32^ Currently, countries where malaria is endemic rely on RDTs to screen for malaria.^33^ Our algorithm achieved a 99.5% sensitivity and a 90.0% specificity at high-sensitivity operating points, whereas standard RDTs achieved sensitivities of 80% to 95% and specificities of 85% to 99%.^32,34^ A possible explanation of our algorithm’s superior performance might be that a *P falciparum*–infected cell in thin smears could provide more details for a deep learning algorithm to identify compared with thick smears. Compared with RDTs, which lack information regarding parasite density and life-cycle stages, our framework not only provided comparable malaria detection performance but also may affect clinical management through automated parasite density estimation, providing pivotal information on disease stratification and treatment response monitoring.^34^ Manual counting of parasites is labor intensive and impractical in resource-constrained settings. With the use of our algorithm, it might be feasible to derive quantitative estimates of parasite density using methods similar to those described in the World Health Organization guideline^3^ but without human intervention. In this manner, practitioners can rapidly stratify patients by severity, optimize therapies, and monitor a patient’s therapeutic response.^31,34^

### Limitations

This study has limitations. First, false-negative detection occurred in several challenging situations, for example, when the cytoplasm and nuclei of parasites were obscure or deformed. False-positive detections happened when stained platelets or impurities on top of RBCs mimic the parasites. To perform better in these challenging scenarios, algorithm training with high-quality images focused on those situations would be helpful. Second, the algorithm was developed to identify only *P falciparum* without other species (eg, *Plasmodium vivax* or *Plasmodium ovale*). Nonetheless, the algorithm is expected to be expanded without difficulty to identify the ring form of other species with tuning because *Plasmodium* species are morphologically similar. Third, our data sets were established from a limited number of patients, which might affect the algorithm’s generalizability. However, the variability in our data sets that resulted from different imported countries, clinical settings, and staining methods might prevent overfitting and help with generalizability. Our clinical validation that mimicked a clinical scenario also showed promising performance for the patients not included in the development set and the potential for real-world application. Nevertheless, how to link negative malaria detection at the cell and image levels with the exclusion of a malaria diagnosis would be another issue for clinical decision making. To further validate and implement the algorithm in real-world settings, work on the clinical workflow design and pilot field validation in countries where malaria is endemic will be required.

## Conclusions

In this study, we built a publicly available benchmark image data set of malaria thin blood smears with reliable annotations (TIME) and demonstrated the potential to develop a deep learning–based malaria detection algorithm with expert-level performance. Both the data sets and algorithm may help accelerate the development of automated microscopic diagnosis and a decision support system in resource-limited countries with heavy malaria burden.

## Notes

Supplement. **eMethods 1.** Participants and Clinical Sample Collection **eMethods 2.** Annotation Guideline **eMethods 3.** Bounding Box Matching or Algorithm Evaluation **eFigure 1.** The F1 Score in Different IoU Thresholds **eFigure 2. **The Histogram of Number of Malaria-Infected Cells per Image **eFigure 3.** Comparison of Annotations in the Reliability Test Subset **eFigure 4. **Comparison of Annotations in the Clinical Validation Set **eFigure 5. **Comparison of Detection Results by Algorithm and Individual Microscopists With Reference Standard at Cell Level in the Clinical Validation Set **eFigure 6. **Comparison of Detection Results by Algorithm and Individual Microscopists With Reference Standard at Image Level in the Clinical Validation Set
