# Supplementary material for: Assessment of Expert-Level Automated Detection of Plasmodium falciparum in Digitized Thin Blood Smear Images
Source: JAMA Netw Open. 2020 Feb 28;3(2):e200206. doi: 10.1001/jamanetworkopen.2020.0206 (PMC7049085; doi:10.1001/jamanetworkopen.2020.0206)
Supplement: Supplement. — eMethods 1. Participants and Clinical Sample Collection eMethods 2. Annotation Guideline eMethods 3. Bounding Box Matching or Algorithm Evaluation eFigure 1. The F1 Score in Different IoU Thresholds eFigure 2. The Histogram of Number of Malaria-Infected Cells per Image eFigure 3. Comparison of Annotations in the Reliability Test Subset eFigure 4. Comparison of Annotations in the Clinical Validation Set eFigure 5. Comparison of Detection Results by Algorithm and Individual Microscopists With Reference Standard at Cell Level in the Clinical Validation Set eFigure 6. Comparison of Detection Results by Algorithm and Individual Microscopists With Reference Standard at Image Level in the Clinical Validation Set [file jamanetwopen-3-e200206-s001.pdf]

Supplementary Online Content

Kuo P-C, Cheng H-Y, Chen P-F, et al. Assessment of expert-level automated detection of *Plasmodium falciparum* in digitized thin blood smear images. *JAMA Netw Open*. 2020;3(2):e200206. doi:10.1001/jamanetworkopen.2020.0206

eMethods 1. Participants and Clinical Sample Collection

eMethods 2. Annotation Guideline

eMethods 3. Bounding Box Matching or Algorithm Evaluation

eFigure 1. The F1 Score in Different IoU Thresholds

eFigure 2. The Histogram of Number of Malaria-Infected Cells per Image

eFigure 3. Comparison of Annotations in the Reliability Test Subset

eFigure 4. Comparison of Annotations in the Clinical Validation Set

eFigure 5. Comparison of Detection Results by Algorithm and Individual Microscopists With Reference Standard at Cell Level in the Clinical Validation Set

eFigure 6. Comparison of Detection Results by Algorithm and Individual Microscopists With Reference Standard at Image Level in the Clinical Validation Set

This supplementary material has been provided by the authors to give readers additional information about their work.

© 2020 Kuo P-C et al. *JAMA Network Open.*

eMethods 1. Participants and clinical sample collection

Malaria is a notifiable disease in Taiwan. Every suspected malaria patient would be reported to Taiwan Centers for Disease Control.The patients enrolled in this study were selected from all the confirmed malaria patients from 2003 to 2018. The patients traveled back from at least 9 different countries, mostly from Africa and Southeast Asia (Nigeria, Kenya, Ghana, Liberia, Sierra Leone, South Africa, Guinea, Vietnam, and Indonesia), and were notified from at least 16 different hospitals around Taiwan. Data on relevant comorbidities could not be retrospectively retrieved. The diagnoses of all malaria-positive patients were confirmed by PCR test. All were *Plasmodium falciparum* infection.

Blood samples were collected either before, during, after the treatment, or during the post-treatment follow-up period, which was up to one year according to the working protocol of Taiwan CDC. All blood smears enrolled in our data sets were thin smears made before the start of treatment and followed the WHO guideline. Staining technique used was mainly Giemsa stain, with some Liu's stain and Wright-Giemsa stain. All the smear slides used in this study were stained in Taiwan CDC and thus we could guarantee the smears were all made following the same protocol and their quality.

eMethods 2. Annotation guideline

Target of annotation
Bounding boxes are annotated on digitized thin blood smear images for: 1. Confirmed malaria-infected red blood cells, and 2. Highly-suspected malaria-infected blood cells.

Steps of Annotation
The bounding box of a malaria-infected red blood cell is annotated by following the steps below:

1. Bounding box annotation
   The criteria of creating a bounding box for malaria-infected cell were as follows:
   - Find an infected blood cell including a parasite whose characteristics were identifiable, such as containing nucleus and cytoplasm.
   - Create a bounding box to include the whole infected blood cell. The box should be as close to the exact size of the cell as possible.
   - If the infected blood cell is overlapped with other blood cells, the bounding box should cover the most possible area containing the whole infected blood cell.
2. Confirm the label for the bounding box
   - After creating the bounding box, each box is labelled according to the species and life-cycle stage of the infecting malaria parasite.
   - Labels included the species of malaria and four different life-cycle stages of *P. falciparum* (ring form/trophozoites, schizont, gametocyte), for example, *P. falciparum*/ring form.
   - The label “Indeterminate” is used when the blood cell is suspicious for infection but could not be confirmed with full confidence.

Process of majority decision and adjudication
To generate reference standard of highest quality possible for the clinical validation set, a process of majority decision and discussion among the three annotating experts were carried out for adjudication. After the three experts of Taiwan CDC annotated the 500 images in the clinical validation set individually, majority decision of boxes and labels made by different experts were made as follows: bounding boxes annotated by different experts were matched by the same algorithm described in the Algorithm Evaluation of the discussion section. Boxes with intersection-over-union (IoU) greater than 0.3 were grouped and considered as annotating the same malaria-infected cell. For each cell, the union of the matched boxes was taken to be the ground truth bounding box of the infected cell when it was annotated by two or more experts. A majority vote among the labels of the matched boxes by different experts was utilized to confirm the ground truth label of the life-cycle stage. When the result of majority vote was inconclusive, for example, when three experts labeled different life-cycle stage for the same infected cell, or when two experts labeled the infected cell with indeterminate, a second round of review would be conducted by the panel of the three experts. The three experts held a group discussion for adjudication regarding the life-cycle stages of the inconclusive boxes. The resulting consensus from the process of matching, majority decision, and adjudication served as the reference standard of malaria diagnosis, against which the performances of our algorithm and four external clinical laboratory scientists were evaluated.

© 2020 Kuo P-C et al. *JAMA Network Open.*

eMethods 3. Bounding box matching for algorithm evaluation

The matching algorithm is used to determine whether a predicted bounding box (identified as an infected cell by our algorithm) matched any ground truth box (confirmed as an infected cell by the experts). Each predicted bounding box would be paired with, if existed, a most overlapped ground truth counterpart with their intersection over union (IoU) greater than 0.3. The IoU threshold of 0.3 was determined to maximize the performance of malaria detection in our task: To properly measure the performance of any object detection algorithms, a threshold of intersection-over-union (IoU) must be determined in advance. For example, in PASCAL VOC2007 challenge, IoU threshold of 0.5 was predefined, while for the COCO 2017 challenge (http://cocodataset.org/#detection-2017), 10 IoU thresholds from 0.5 to 0.95 were used to calculate mean average precision (mAP) among 80 object categories. Since there is little information about how to select IoU threshold for the task of detecting malaria infection from previous works, we first studied the effect of different IoU threshold on matching bounding boxes, by examining inter-rater reliability at cell level in the reliability test subset. In eFigure 1, we could find the F1 scores on bounding box matching increase as IoU threshold decrease from 0.9 to 0.4, and reach plateaus when IoU threshold further decreases. Also taking into consideration P. falciparum parasites range in size from 1 to 20 microns in diameter, while a normal red blood cell has a diameter of 7 microns, IoU of 0.3 is selected for matching bounding boxes in our project.

If matched, the predicted labels (species and life-cycle stages) of the box is then compared with the ground truth class of the ground truth box. If they are identical, the predicted box is considered as true positive. False positive is defined when the predicted bounding box is not matched to any ground truth box. False negative includes two scenario: First, when a ground truth box is matched to a predicted box but the class is not identical; second, when a ground truth box is not matched to any predicted box. Since there is no ground truth box for non-infected cells, there is no true negative existed at the cell level. The numbers of true positive, false positive, and false negative predictions were then used to calculate precision-recall curves with average precision and free-response receiver operating characteristic (FROC) curves.

© 2020 Kuo P-C et al. *JAMA Network Open.*

eFigure 1. The F1 score in different IoU thresholds.

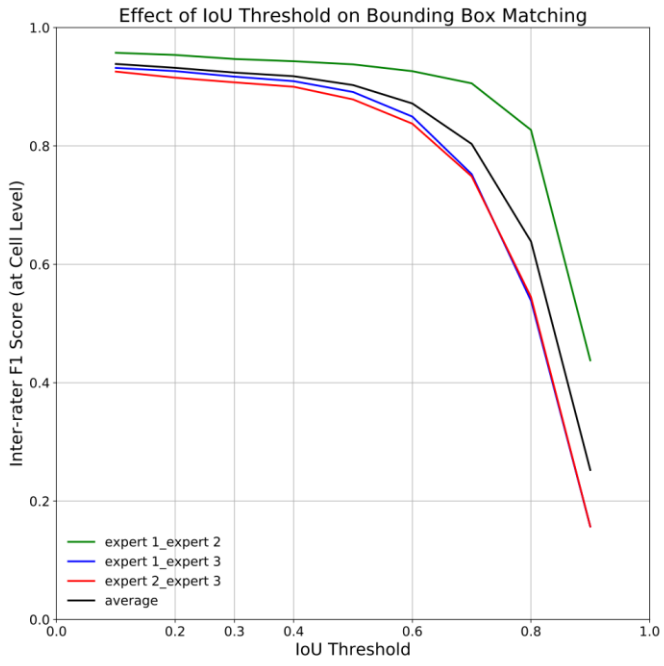

© 2020 Kuo P-C et al. *JAMA Network Open.*

eFigure 2. The histogram of number of malaria-infected cells per image.

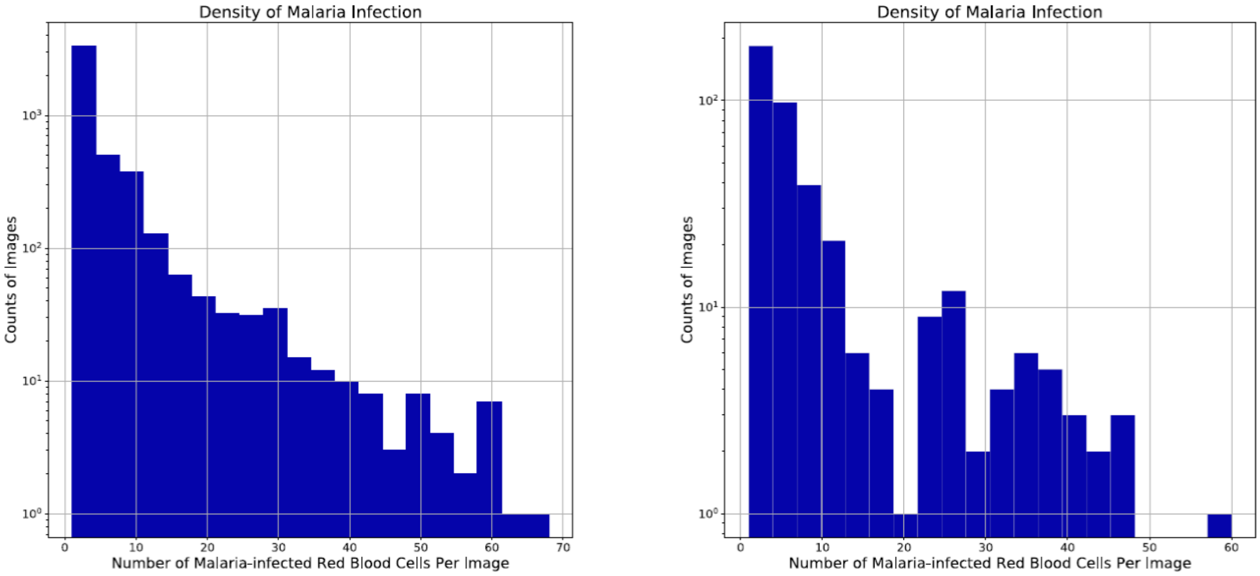

A) Development set

B) Clinical validation set

© 2020 Kuo P-C et al. *JAMA Network Open.*

eFigure 3. Comparison of annotations in the reliability test subset.

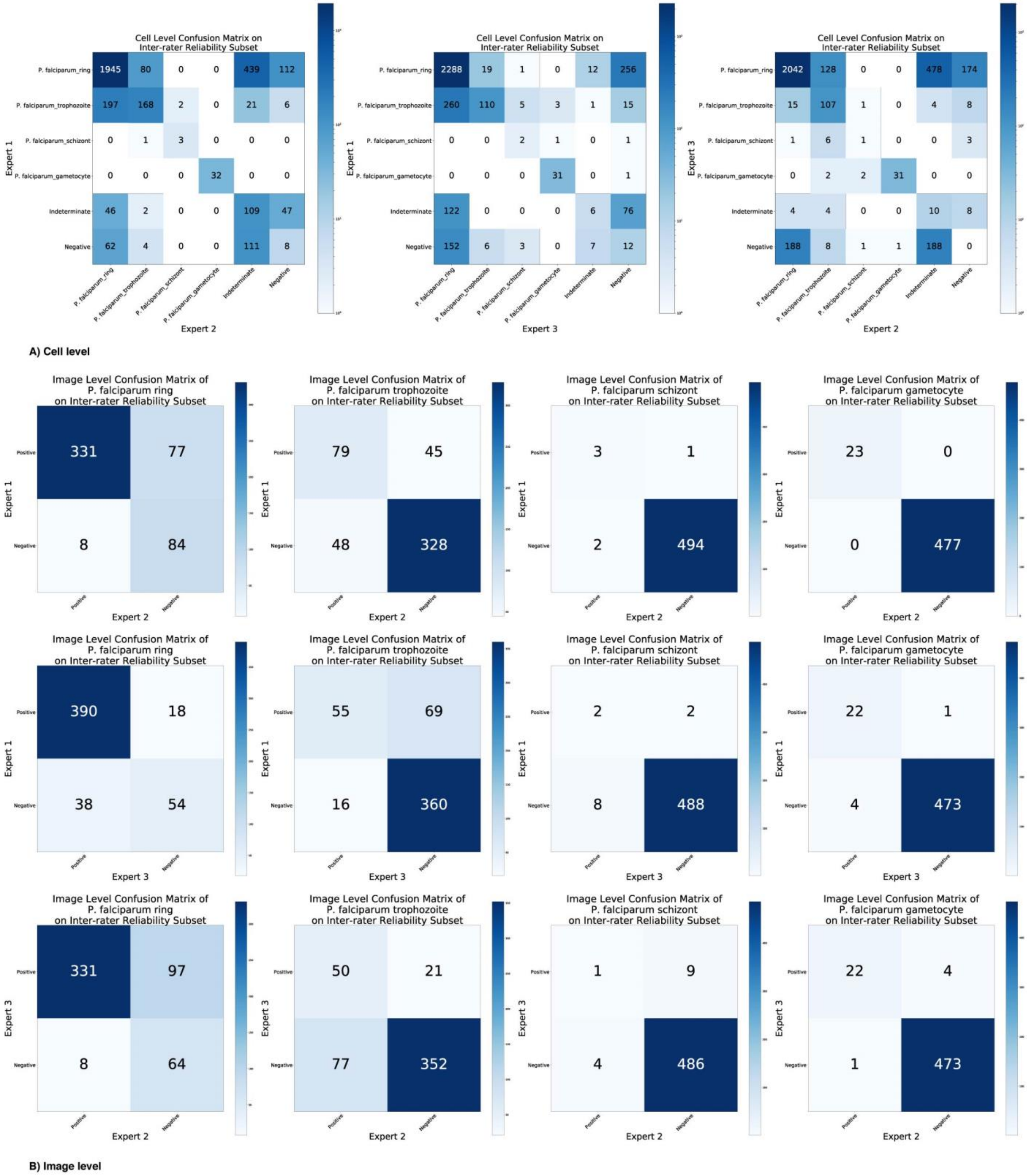

Confusion matrices comparing annotations of each pair of experts rating the reliability test subset are presented.

© 2020 Kuo P-C et al. *JAMA Network Open.*

eFigure 4. Comparison of annotations in the clinical validation set.

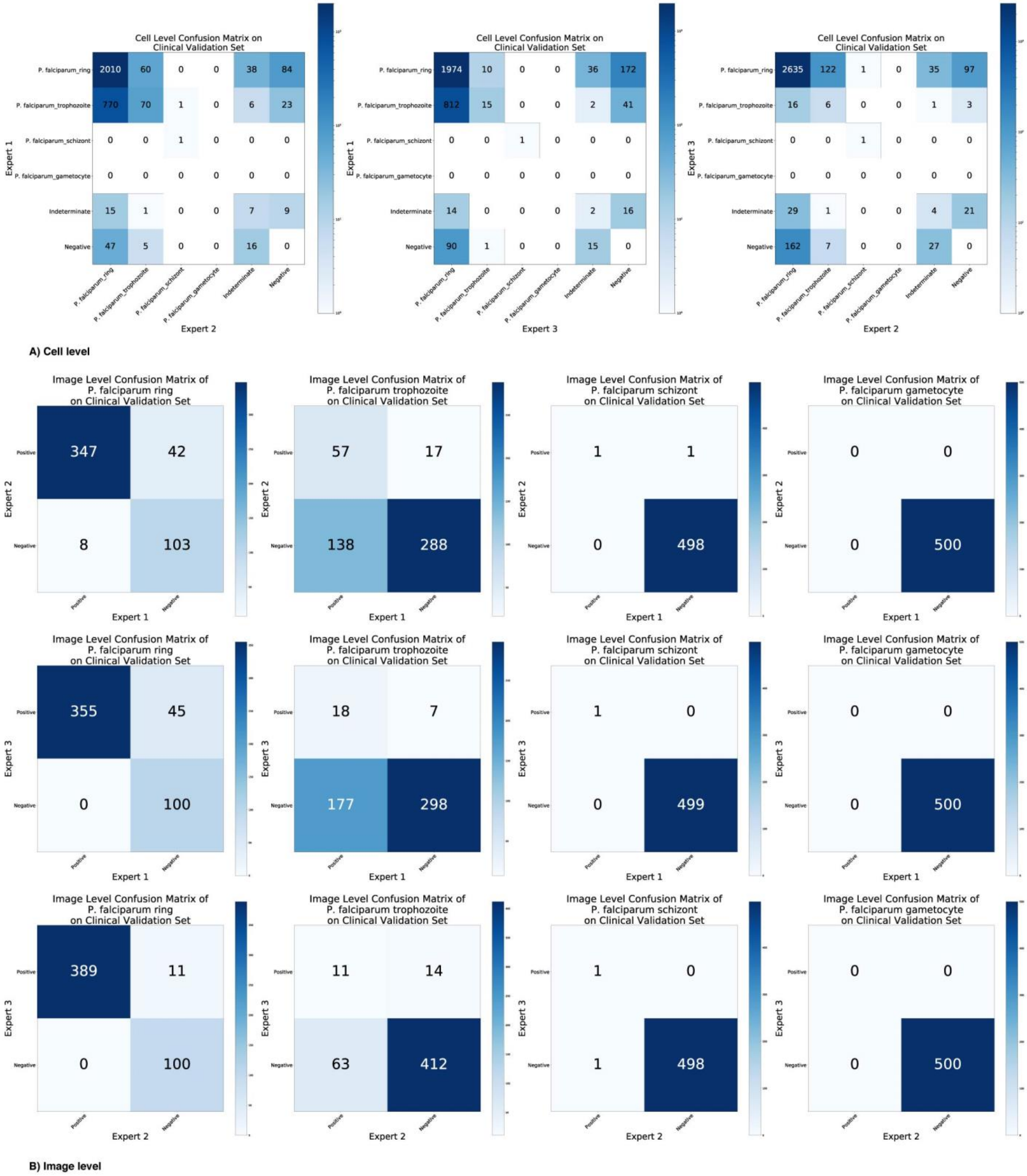

(A) Cell level (B) Image level. Confusion matrices comparing annotations of each pair of experts rating the clinical validation set are presented.

© 2020 Kuo P-C et al. *JAMA Network Open.*

eFigure 5. Comparison of detection results by algorithm and individual microscopists with reference standard at cell level in the clinical validation set.

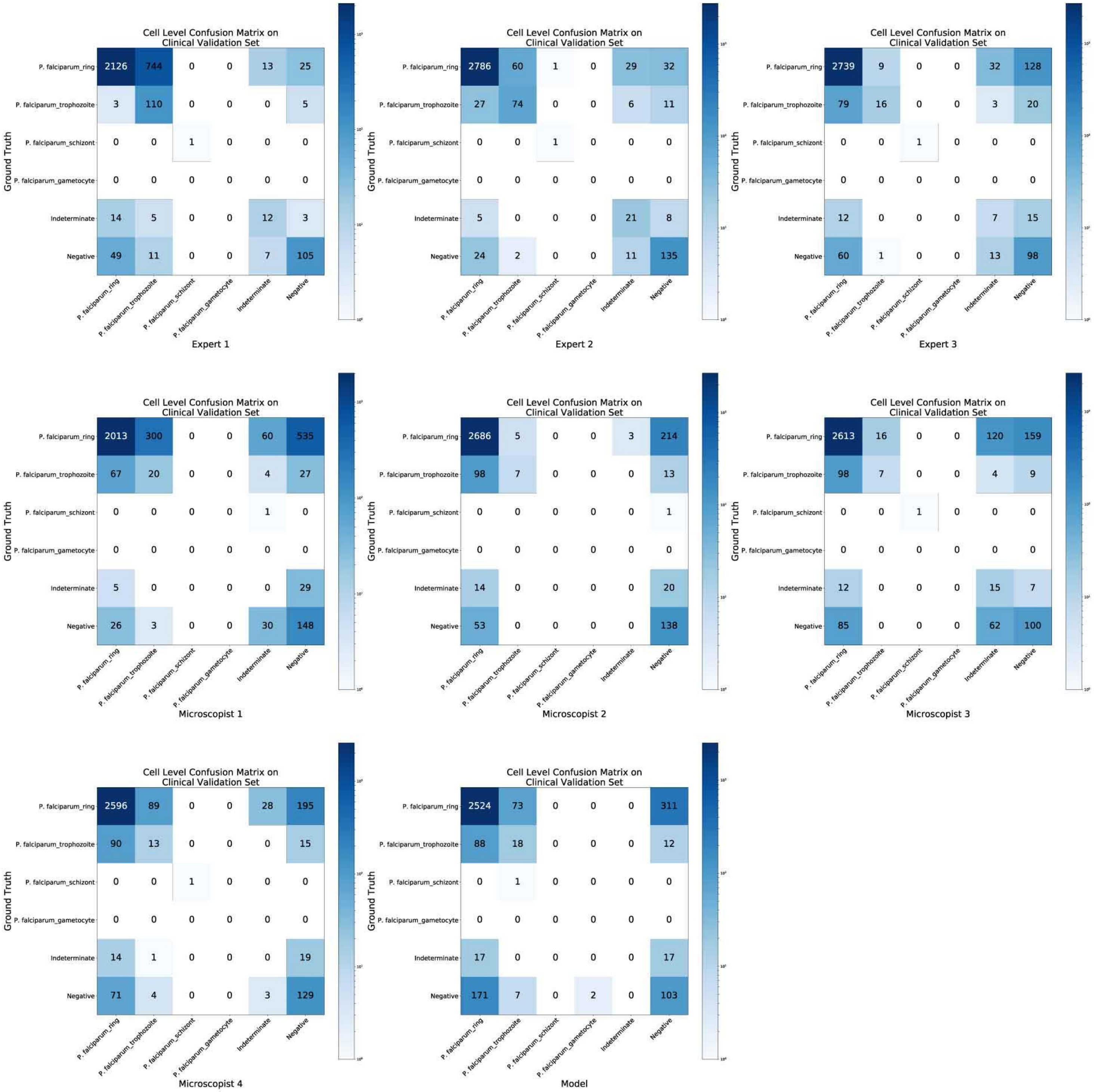

Confusion matrices comparing detection results by algorithm and individual microscopists with reference standard in the clinical validation set are presented at cell level.

© 2020 Kuo P-C et al. *JAMA Network Open.*

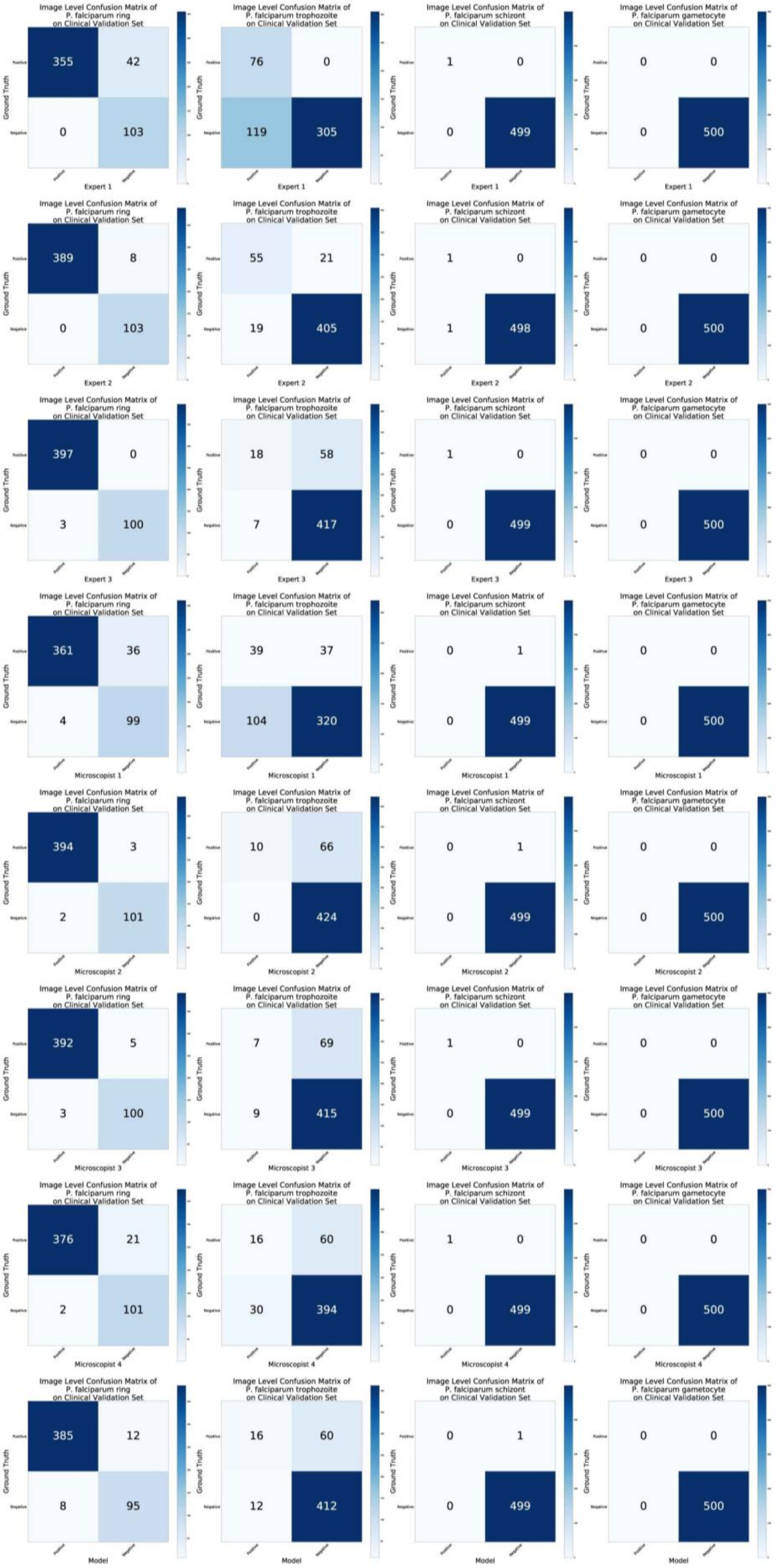

eFigure 6: Comparison of detection results by algorithm and individual microscopists with reference standard at image level in the clinical validation set.

Confusion matrices comparing detection results by algorithm and individual microscopists with reference standard in the clinical validation set are presented at image level.

© 2020 Kuo P-C et al. *JAMA Network Open.*
